# Supplementary material for: Fate mapping analysis reveals a novel murine dermal migratory Langerhans-like cell population
Source: eLife. 2021 Mar 26;10:e65412. doi: 10.7554/eLife.65412 (PMC8110305; doi:10.7554/eLife.65412)
Supplement: Transparent reporting form [file elife-65412-transrepform.docx]

***eLife’s* transparent reporting form**

We encourage authors to provide detailed information *within their submission* to facilitate the interpretation and replication of experiments. Authors can upload supporting documentation to indicate the use of appropriate reporting guidelines for health-related research (see [EQUATOR Network](http://www.equator-network.org/%20)), life science research (see the [BioSharing Information Resource](https://biosharing.org/)), or the [ARRIVE guidelines](http://www.plosbiology.org/article/info:doi/10.1371/journal.pbio.1000412) for reporting work involving animal research. Where applicable, authors should refer to any relevant reporting standards documents in this form.

If you have any questions, please consult our Journal Policies and/or contact us: [editorial@elifesciences.org](mailto:editorial@elifesciences.org).

**Sample-size estimation**

- You should state whether an appropriate sample size was computed when the study was being designed
- You should state the statistical method of sample size computation and any required assumptions
- If no explicit power analysis was used, you should describe how you decided what sample (replicate) size (number) to use

Please outline where this information can be found within the submission (e.g., sections or figure legends), or explain why this information doesn’t apply to your submission:

No specific power analysis was used. Sample sizes were chosen based on our experience with similar experiments (a minimum of 4–6 mice) and based on the availability of age-matched mice.

**Replicates**

- You should report how often each experiment was performed
- You should include a definition of biological versus technical replication
- The data obtained should be provided and sufficient information should be provided to indicate the number of independent biological and/or technical replicates
- If you encountered any outliers, you should describe how these were handled
- Criteria for exclusion/inclusion of data should be clearly stated
- High-throughput sequence data should be uploaded before submission, with a private link for reviewers provided (these are available from both GEO and ArrayExpress)

Please outline where this information can be found within the submission (e.g., sections or figure legends), or explain why this information doesn’t apply to your submission:

Each flow cytometry sample was obtained from one individual mouse or from pooled cells obtained from 2-3 distinct mice of the same mouse strain, in case the cell number was insufficient for a comprehensive flow cytometry analysis.

For scRNAseq analysis dermal cells were isolated from six C57/BL6 mice were pooled for further processing (Fig. 2). For transcriptome analysis of CD45.1^+^ and CD45.2^+^ LC^like^ cells, each sample was obtained from ten pooled mice (Fig. 5E).

**Statistical reporting**

- Statistical analysis methods should be described and justified
- Raw data should be presented in figures whenever informative to do so (typically when N per group is less than 10)
- For each experiment, you should identify the statistical tests used, exact values of N, definitions of center, methods of multiple test correction, and dispersion and precision measures (e.g., mean, median, SD, SEM, confidence intervals; and, for the major substantive results, a measure of effect size (e.g., Pearson's r, Cohen's d)
- Report exact p-values wherever possible alongside the summary statistics and 95% confidence intervals. These should be reported for all key questions and not only when the p-value is less than 0.05.

Please outline where this information can be found within the submission (e.g., sections or figure legends), or explain why this information doesn’t apply to your submission:

All statistical tests are fully described in detail in the Figure Legends. Samples were analyzed by two-tailed Student's *t*-test to determine statistical differences between two groups. A two-way ANOVA with Bonferroni-post-test was used to determine the differences between more than two groups. A P value <0.05 was considered to be statistically significant. The number of animals used per group is indicated in the Figure Legends as ‘‘n.’’

**Group allocation**

- Indicate how samples were allocated into experimental groups (in the case of clinical studies, please specify allocation to treatment method); if randomization was used, please also state if restricted randomization was applied
- Indicate if masking was used during group allocation, data collection and/or data analysis

Please outline where this information can be found within the submission (e.g., sections or figure legends), or explain why this information doesn’t apply to your submission:

Not applicable

**Additional data files (“source data”)**

- We encourage you to upload relevant additional data files, such as numerical data that are represented as a graph in a figure, or as a summary table
- Where provided, these should be in the most useful format, and they can be uploaded as “Source data” files linked to a main figure or table
- Include model definition files including the full list of parameters used
- Include code used for data analysis (e.g., R, MatLab)
- Avoid stating that data files are “available upon request”

Please indicate the figures or tables for which source data files have been provided:

All RNA-sequencing data have been deposited in the Gene Expression Omnibus public database under accession number GSE139877 (Fig. 5). Single cell RNAseq have been deposited into NCBI SRA database with BioProject ID: PRJNA625270 (Fig. 2). Source data are included to Fig. 4, 5, 6, 7 and 8.
